# D347G in PA is critical for the pathogenicity of H9N2 avian influenza A virus in mice

**DOI:** 10.1080/21505594.2026.2711518

**Published:** 2026-08-02

**Authors:** Zhehong Zhao, Ye Tian, Wenjie Jiang, Xuefeng Yin, Xinran Chu, Quan Xie, Tuofan Li, Hongxia Shao, Aijian Qin, Jianqiang Ye, Zhimin Wan

**Affiliations:** a Key Laboratory of Jiangsu Preventive Veterinary Medicine, Key Laboratory for Avian Preventive Medicine, Ministry of Education, College of Veterinary Medicine, Yangzhou University, Yangzhou, Jiangsu, China; b Jiangsu Co-Innovation Center for Prevention and Control of Important Animal Infectious Diseases and Zoonoses, Yangzhou, Jiangsu, China; c Joint International Research Laboratory of Agriculture and Agri-Product Safety, The Ministry of Education of China, Yangzhou University, Yangzhou, Jiangsu, China; d Institute of Agricultural Science and Technology Development, Yangzhou University, Yangzhou, Jiangsu, China; e China Institute of Veterinary Drug Control, Beijing, China

**Keywords:** H9N2, pathogenicity, mice, PA, D347G mutation, polymerase activity

## Abstract

Some subtypes of avian influenza A viruses (IAVs) have been associated with human infections (e.g. H3N8, H5Ny, H7Ny, H9N2, and H10Ny), and the ability of these viruses to cause zoonotic infections further increases the public health risk of avian IAVs. Among them, H9N2 virus is one of particular importance, both in its own right and as a contributor of internal gene segments to other emerging zoonotic avian IAVs. In recent years, an increasing number of H9N2 strains have been observed to be highly pathogenic in mice without prior adaptation. In this study, we found that a naturally occurring H9N2 isolate, A/chicken/Jiangxi/198/2019 (JX198), is lethal in mice. To investigate the molecular basis for the high virulence of JX198 in mice, a series of reassortants and mutants were generated and tested. We found that the PA, especially D347G mutation in PA, is responsible for the increased pathogenicity of JX198 in mice. Notably, D347G in PA of JX198 significantly alters the polymerase activity, vRNA production, and plaque-formation. Thus, our data demonstrate that a novel molecular marker, D347G in PA, determines the virulence of H9N2 in mice, posing a potential risk of H9N2 with D347G in PA for public health and highlighting the significance of continuing surveillance of H9N2 field strains.

## Introduction

Influenza A virus (IAV) is a negative-sense, single-stranded RNA virus, containing 8 gene segments, which encode 8 structural proteins and several non-structural proteins, posing a threat to global health through seasonal epidemics and recurring pandemics [1]. Avian species are the natural reservoir of IAVs, and avian IAVs are the major source of novel IAV infections in humans, leading to a new pandemic. However, avian IAVs require some mutations to efficiently replicate and transmit in humans, especially in the viral RNA-dependent RNA polymerase (vRdRp) [2,3]. The vRdRp is comprised of three components (PB2, PB1, and PA) that bind to viral RNAs which are encapsulated by NP protein to form the viral ribonucleoprotein (vRNP) complex [4]. Replication and transcription of the IAV genome in the nuclei of infected cells are mediated by vRNP. Some mutations in vRdRp, which increase the polymerase activity of avian IAV, are necessary for the adaptation of avian IAV to mammalian hosts. In particular, the single mutation E627K in PB2 of several subtypes of avian IAVs has been shown to significantly increase the activities of avian vRdRp in mammalian cells, leading to greater replication and pathogenicity in mammals [5–7].

H9N2 virus belongs to low-pathogenic avian influenza virus (LPAIV), which is enzootic in poultry across Asia, Africa, and the Middle East [8,9]. H9N2 virus causes mild disease in poultry, but can cause high mortality if co-infecting with other pathogens, resulting in a significant economic burden on the poultry industry [10,11]. In addition, H9N2 also poses a threat to public health as zoonotic diseases are being reported in different countries, as evidenced by numbers confirmed human infection cases [12]. It is worth noting that H9N2 virus has provided internal genes for some subtypes of avian IAV that infects humans in recent decades, including H5Ny, H7N9, H10N8, H10N3, and H3N8 [13–17].

Since H9N2 virus was first isolated from chickens in Guangdong Province in 1994, it has evolved into multiple lineages in China, represented by A/chicken/Beijing/1/1994 (BJ/94-like), A/quail/Hong Kong/G1/1997 (G1-like), A/chicken/G9/1997 (G9-like), and A/chicken/Shanghai/F/1998 (F/98-like) [18,19]. Moreover, different representative strains of H9N2 viruses have undergone continuous reassortment [20,21]. A novel genotype of H9N2 virus (G57, also known as genotype S) emerged in chickens in eastern China in 2007 and has become the dominant genotype throughout China since 2010, with the PB2 and M genes being derived from G1-like viruses and the other genes from Y280-like viruses [14,22,23]. Although H9N2 virus belongs to LPAIV, an increasing number of H9N2 viruses isolated from China were highly pathogenic in mice [24,25]. Amino acid variations in the PA subunit have been implicated in the adaptation of H9N2 viruses to mice, with position 347 identified as a critical residue in mouse-adapted strains generated by serial *in vivo* passing [26,27]. However, whether naturally occurring mutations at position 347 of the PA protein contribute in high pathogenicity of field H9N2 isolates in mice without prior adaptation remains largely unknown. In this study, we isolated a naturally circulating G57 genotype H9N2 virus from chickens in China, A/chicken/Jiangxi/198/2019 (JX198), that exhibits high lethality in mice. We confirmed that the vRNP complex is responsible for the increased pathogenicity of JX198, especially PA D347G is the major virulence determinant of JX198. This finding demonstrates that the PA D347G mutation has already emerged in naturally prevalent H9N2 field strains, highlighting the necessity of continuous surveillance for this mutation in AIV monitoring to assess potential zoonotic risks.

## Materials and methods

### Viruses and cells

Two H9N2 strains of JX198 and A/chicken/Xuzhou/508/2019(H9N2) (XZ508) used in this study were isolated from chickens in 2019. Reassortant H9N2 viruses were rescued using reverse genetics [28]. All viruses were propagated in 9-day-old specific pathogen-free (SPF) embryonated chicken eggs and stored at −80 ºC. Madin-Darby canine kidney (MDCK) and human embryonic kidney 293T cells were maintained in Dulbecco’s modified Eagle’s medium (DMEM), containing 10% fetal calf serum (Lonsera, Shanghai, China) and 1% antibiotic.

### Genomic sequencing and phylogenetic analyses of the viruses

Each segment of JX198 and XZ508 was sequenced and cloned into plasmid of pDP2002 according to previous report [29]. Briefly, viral RNAs of JX198 and XZ508 were extracted from allantoic fluid and cDNAs were synthesized by reverse transcription (RT) with the primer: 5’-AGCGAAAGCAGG-3,’ and all 8 segments were amplified by RT-PCR with specific primers. The linearized influenza vector pDP2002 was also amplified. The PCR amplicon for each segment was recombined with the linearized pDP2002 vector using the Exnase^TM^ II (Vazyme Biotech, Nanjing, China) according manufacturer’s instructions. The constructed plasmids were sequenced by Sanger sequencing (Jinweizhi Biotechnology, Suzhou, China). For phylogenetic analysis, the sequences of the H9N2 reference strains were downloaded from Influenza Virus Database of National Center for Biotechnology Information (NCBI). Phylogenetic trees were constructed by the maximum likelihood method in MEGA 11.

### Generation of viruses by reverse genetics

A pair of reassortant H9N2 viruses used in this study were generated by reverse genetics as previously described [28]. Briefly, eight plasmids (1 µg of each plasmid) were co-transfected into co-cultured 293T and MDCK cells by TransIT®-LT1 Transfection Reagent (Mirus Bio LLC, Madison, WI, USA). After 6 h incubation, the medium was replaced with 1 mL of fresh opti-MEM. After 18 h incubation, the medium was replaced with 2 mL of fresh opti-MEM medium with 1 µg/mL TPCK-Trypsin. At day 3 post-transfection, the supernatants were collected and confirmed by hemagglutination (HA) assay and then inoculated into SPF embryonated chicken eggs.

### Site-directed mutagenesis

Nucleotide changes corresponding to amino acid mutations were introduced into PA gene in plasmid pDP2002 with the Mut Express II Fast Mutagenesis Kit V2 (Vazyme Biotech, Nanjing, China), and mutation plasmids were sequenced to verify the presence of indicated mutations.

### Virus plaque assays

All plaque assays were performed in MDCK cells using 1% agar overlay. Briefly, confluent cells in 6-well plates were infected with virus dilutions for 2 h at 37°C. Cells were washed two times with PBS and then overlaid with opti-MEM containing 1% agar and 1 µg/mL TPCK-trypsin. The plates were then incubated at 37°C and 5% CO2. After 72 h incubation, the overlays were removed and the cells were stained with 0.1% crystal violet solution.

### Luciferase assay for polymerase activity

Polymerase activity was measured using mini-genome assay as previously described [30]. Briefly, 293T cells were co-transfected with a luciferase reporter plasmid p-Luci plasmid together with RNP genes from JX198 and/or XZ508 and internal control Renilla plasmid. After 48 h, the luciferase activity was measured using a dual-luciferase reporter system (Vazyme Biotech, Nanjing, China).

### Quantitation of viral vRNA and mRNA

The relative expression levels of viral mRNA and vRNA were determined in various recombinant virus-infected MDCK cells at a multiplicity of infection (MOI) of 1. Total RNA was extracted from the infected MDCK cells at 6, 12, and 24 hours post infection (hpi). For the detection of viral mRNA and vRNA, oligo dT and U12 primers were used to generate cDNAs of mRNA and vRNA. Quantitative PCR (qPCR) for detecting the *NP* gene was performed with the specific primers (Table 1). The expression values of each gene relative to GAPDH were calculated using the 2−ΔΔCT method.

**Table 1.** The specific primer sequences used for detecting vRNA and mRNA.

Target fragment	Orientation	Primer sequence (5”−3”)
GAPDH	Forward	GAGTCAACGGATTTGGTCGT
	Reverse	GACAAGCTTCCCGTTCTCAG
Influenza vRNA amplification for RT step	/	GGCCGTCATGGTGGCGAATCGTTCGTCACATCTCATCTACCTC
qPCR primers for influenza vRNA	Forward	GGCCGTCATGGTGGCGAAT
	Reverse	CCACACCCTTAGGTAACCCAGTAGA
Influenza mRNA amplification for RT step	/	CCAGATCGTTCGAGTCGTTTTTTTTTTTTTTTTTCT
qPCR primers for influenza mRNA	Forward	CCAGATCGTTCGAGTCGT
	Reverse	AAAAAGAGATCGTGGATTACGTCGC

## Western blot

MDCK cells were infected with each H9N2 virus at an MOI of 1. The infected cell samples were collected and lysed at 6, 12, and 24 hpi to obtain total cellular protein lysates. The lysates were denatured at 98°C for 5 minutes in protein loading buffer, followed by electrophoretic separation using 10% SDS-PAGE. Then, separated proteins were transfected onto nitrocellulose membranes. After blocking with block buffer, the membranes were co-incubated with monoclonal antibodies (MAbs) against influenza virus NP protein and GADPH for 2 h at room temperature (RT). GADPH was used as an internal reference protein. After washing 3 times with PBST, the membrane was incubated with horseradish peroxidase (HRP)-conjugated goat anti-mouse IgG antibody at RT for 1 h. After washing 5 times with PBST, the immunoreactive bands on the membranes were developed through chemiluminescent reagents.

### Mouse study

Five-week-old BALB/c female mice were purchased from Experiment Animal Center of Yangzhou University (Yangzhou, China). All mice were housed in the standard cages (up to 5 mice/cage). All mice were acclimated for 3 days before the experiments. The mice (11 mice/group) were anesthetized with isoflurane and infected with the reassortant H9N2 viruses at 10^5^ TCID_50_ in a volume 25 µL by intranasal inoculation, respectively. At 3-and 5-day post-infection (dpi), three mice from each group were euthanized, and the lungs were collected for virus titration. Five infected mice per group were monitored daily for body weight loss and clinical signs. The mice with the body weight loss more than 25% will be anesthetized and humanely euthanized. We strictly adhere to the veterinary guidelines of the American Veterinary Medical Association for the anesthesia and euthanasia of mice. During anesthesia, mice are placed in a sealed chamber containing isoflurane (Friends Honesty Life Sciences Company Limited, Hong Kong). Once anesthesia is achieved, lethality is induced via cervical dislocation within 5 to 10 seconds.

### Statistical analysis

The data were analyzed using GraphPad PRISM 8 software (GraphPad Software, La Jolla, CA). The student *t-*test was used to calculate *p* values. All experiments were performed in triplicate, and all data are presented as the means ± SEM. Significant levels were set as follows: *p < 0.05; **p < 0.01; ***p < 0.001; ****p < 0.0001.

### Ethics statement

The mouse studies were performed in accordance with the institutional animal guidelines approved by the Animal Care Committee at Yangzhou University, China. The protocols were approved by the Animal Committee of Yangzhou University with ethical permission number NSFC2023-SYXY-09 (licensed on 23 May 2023).

## Results

### Replication and pathogenicity of JX198 in mice

**Figure 1.**
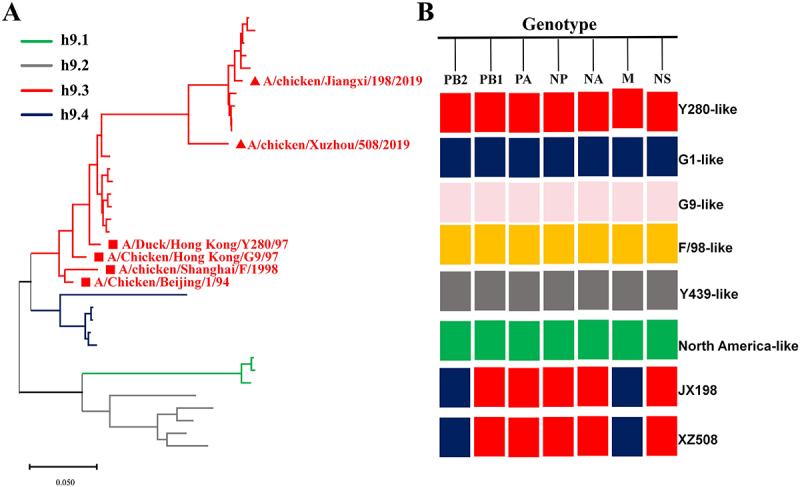
Phylogenetic analyses of J198 and XZ508 viruses. (A) HA phylogenetic tree was constructed with MEGA11 using the maximum likelihood method. The red triangles indicate viruses characterized in this study, and the red squares indicate representative strains of h9.3 lineage. (B) Colored boxes show the lineage classification of each segment of H9N2 viruses belonging to Y280-like, G1-like, G9-like, F/98-like, Y439-like, and North America-like.

**Figure 2.**
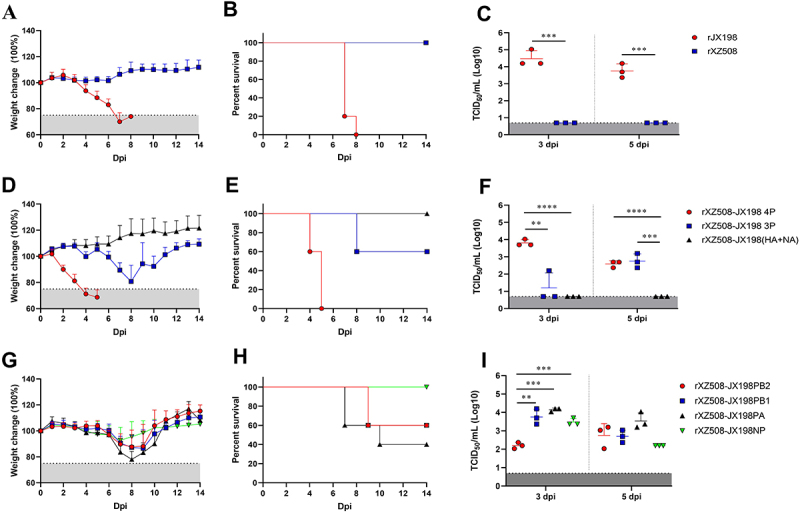
Replication and pathogenicity of JX198 and XZ508 viruses. 5-week-old BALB/c (11 mice/group) were infected with 10^5^ TCID_50_ of rJX198, rXZ508, or their reassortants. (A, D, and G) Percentages of change in body weight and (B, E, and H) survival following infection with each virus. (C, F, and I) Virus titers in lung homogenates collected at 3 and 5 dpi were measured by TCID_50_ in MDCK cells. The *p* values were analyzed by using the student *t*-test. *p < 0.05; **p < 0.01; ***p < 0.001; ****p < 0.0001.

In our initial study, we found the H9N2 isolate JX198, but not XZ508, was lethal to BALB/c mice. To understand the genetics of the JX198 and XZ508, the whole genomes of these two viruses were sequenced and analyzed. A phylogenetic tree based on HA revealed that these two viruses fell into the h9.3 branch and was closed to A/Duck/Hong Kong/Y280/97 (H9N2) (Y280) (Figure 1(A)). Phylogenetic analysis of the other 7 genes showed that PB2 and M genes of the two viruses derived from G1-like and other 5 genes (PB1, PA, NP, NA, and NS) were from Y280-like (Figure 1(B)). These results indicated that JX198 and XZ508 belonged to G57 genotype of H9N2, which has been predominant in chickens in China since 2010 [14]. To characterize the pathogenicity of the two H9N2 viruses in mice, BALB/c were intranasally challenged with wild type (WT) JX198 and XZ508 viruses, respectively. WT JX198 infection induced rapid body weight loss in mice, with all infected mice succumbing or had to be euthanasia by 5 dpi, whereas mice infected with WT XZ508 exhibited no obvious clinical symptoms (Supplementary Figure S1). To further evaluate pathogenicity of these two H9N2 viruses, we rescued JX198 and XZ508 by reverse genetics and performed the mice challenged study. Consistent with the WT strains, the rescued viruses exhibited highly similar pathogenic phenotypes in mice. The mice infected with rJX198 displayed rapid body weight loss and weakness from 3 dpi, and all infected mice died or had to be euthanized at 8 dpi (Figure 2(A,B)). Notably, no clinical signs of disease and only minor weight loss were observed in the mice infected with XZ508, and the infected mice were recovered rapidly from 5 dpi (Figure 2(A,B)). Three infected mice in each group were euthanized at 3 and 5 dpi to determine lung viral titers. As shown in Figure 2(C), the replication of rJX198 could be efficiently detected in the lungs of mice with high viral titers, whereas that of rXZ508 was not detected. To confirm successful infection of mice with rXZ508, we performed an immunofluorescence assay (IFA) on serum samples harvested from the rXZ508 infected mice in 14 dpi. Distinct specific fluorescent signals were still observed when XZ508 infected MDCK cells were incubated with 1:400 diluted serum, which serologically verified rXZ508 successfully infected mice (Supplementary Figure S2). All these demonstrated that JX198, but not XZ508, is highly pathogenic to BALB/c mice.

### vRNP plays a major role in the different pathogenicity between JX198 and XZ508

To identify the virulence determiners in JX198, we rescued three reassortants using XZ508 as the backbone. The first reassortant with PB2, PB1, PA, and NP from JX198 and the other genes from XZ508, designated as rXZ508-JX198 4P, the second reassortant with PB2, PB1, and PA from JX198 and the other genes from XZ508, designated as rXZ508-JX198 3P, the third reassortant with HA and NA from JX198 and the other genes from XZ508, designated as rXZ508-JX198 (HA+NA). Mouse study showed that rXZ508-JX198(HA+NA) similar to rXZ508 did not cause weight loss in infected mice, and could not be detected in lungs of the infected mice (Figure 2(D)). However, the mice infected with rXZ508-JX198 4P showed rapid body weight loss and weakness from 2 dpi, and either succumbed or had to be euthanized at 5 dpi (Figure 2(D,E)). rXZ508-JX198 3P also showed high pathogenicity in mice with weight loss beginning at 4 dpi, and the weight loss of the infected mice reached a peak at 8 dpi (about 20%), and 2 out of 5 mice had weight loss over 25% and had to be euthanized (Figure 2(D,E)). Notably, rXZ508-JX198 4P and rXZ508-JX198 3P could be efficiently detected in lungs of infected mice at 3 and 5 dpi, whereas rXZ508-JX198 (HA+NA) similar with XZ508 could not be not detected in lungs of infected mice (Figure 2(F)). All these demonstrated that the vRNP complex is the major contributor to the pathogenicity of JX198 in mice.

To further identify which gene contributes to the different pathogenicity between JX198 and XZ508, four single-gene reassortants using the XZ508 backbone were rescued, designated as rXZ508-JX198PB2, rXZ508-JX198PB1, rXZ508-JX198PA, and rXZ508-JX198NP. As shown in Figure 2(G), mice infected with these four viruses all showed body weight loss. Notably, rXZ508-JX198NP only caused about 10% bodyweight loss at 7 dpi and then recovered, the other three reassortants caused more bodyweight loss than rXZ508-JX198NP, especially rXZ508-JX198PA, which caused over 20% weight loss in infected mice, and 3 out of 5 mice had to be euthanized (Figure 2(G,H)). The higher pathogenicity of rXZ508-JX198PA was also reflected by higher viral titers in lungs of infected mice at 3 and 5 dpi (Figure 2(I)). These data demonstrated that each gene from the vRNP complex (PB2, PB1, PA, and NP) contributes to the pathogenicity of JX198 in mice, and the PA gene of JX198 plays a vital role in the pathogenesis.

### PA gene of JX198 significantly enhances the polymerase activity of XZ508

**Figure 3.**
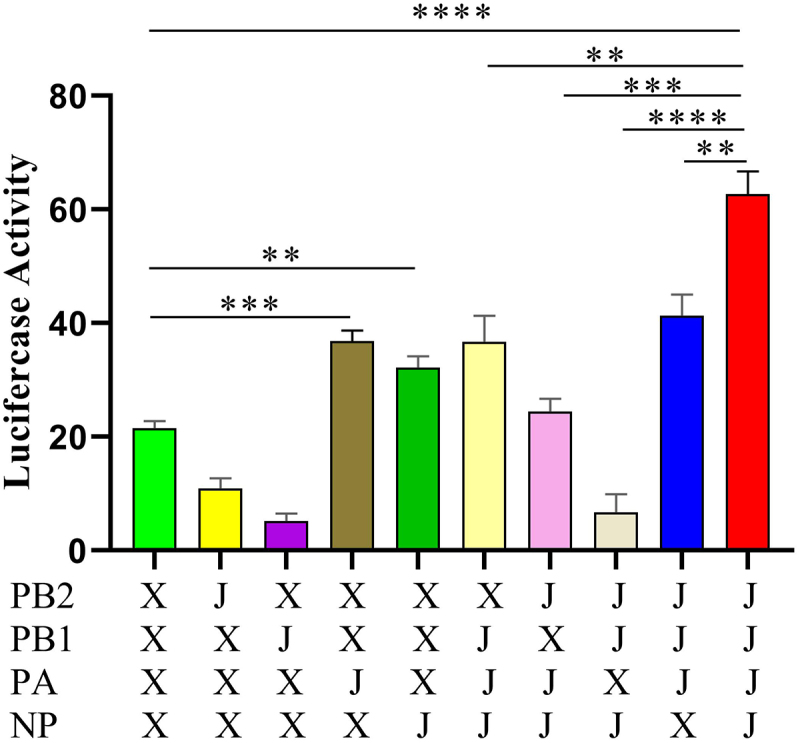
The gene contributes to the difference in the polymerase activity of JX198 and XZ508. Polymerase activity of the vRNP complex of JX198 and XZ508, as well as their corresponding single-gene-segment hybdid derivative, was determined. The experiments were performed with three independent biological replicates. Virus strain abbreviations: J, JX198; X, XZ508. The *p* values were analyzed by using the student *t*-test *p < 0.05; **p < 0.01; ***p < 0.001; ****p < 0.0001.

To investigate whether the pathogenicity of JX198 is associated with the polymerase activity, we conducted a comparison using a minigenome assay with PB2, PB1, PA, and NP genes in 293T cells. As described in Figure 3, the polymerase activity of 4P from JX198 was significantly higher than that of XZ508 (approximately 3-fold). Hybrid vRNP complex assay showed that the polymerase activity of the XZ508 vRNP complex bearing the PA or NP of JX198 was significantly increased compared to that of original XZ508 (Figure 3), whereas that of JX198 vRNP complex bearing PA of XZ508 was significantly decreased compared to that of JX198 (Figure 3). All these demonstrated that PA is the major contributor to the difference of the polymerase activity between JX198 and XZ508.

### D347G in PA plays a significant role in the polymerase activity of JX198

**Figure 4.**
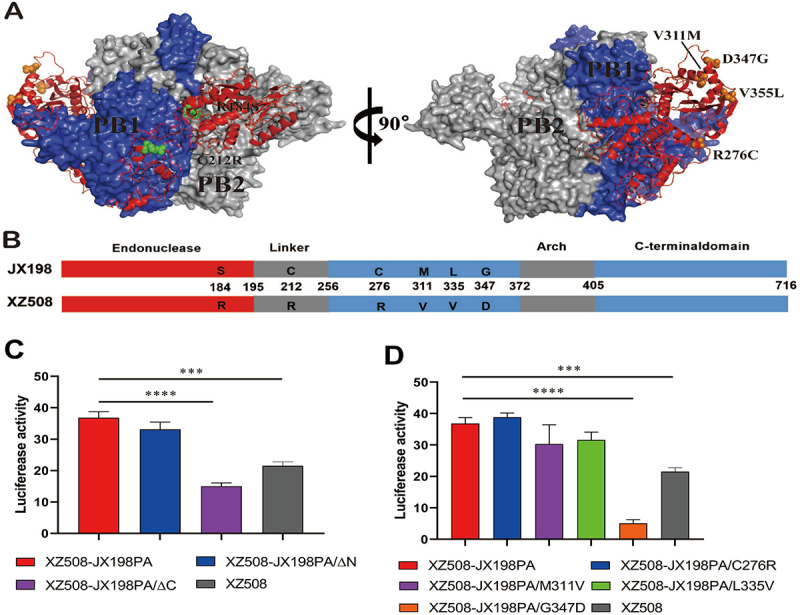
The key amino acid (s) in the PA gene contribute to the difference in the polymerase activity. The six amino acid differences of the PA protein between JX198 and XZ508 are shown in (A) 3D-structure image and (B) schematic sketch. The 3D-stucture image was generated using Pymol software (PDB: 6qnw). (C) and (D) Polymerase activity of vRNP complex of the PA mutations of JX198 with XZ508 backbone. The experiments were performed with three independent biological replicates. The *p* values were analyzed by using the student *t*-test. *p < 0.05; **p < 0.01; ***p < 0.001; ****p < 0.0001.

To investigate the molecular basis of the pathogenicity of PA of JX198, the sequence of PA protein of JX198 and XZ508 was analyzed. Six amino acid variations in the PA protein between JX198 and XZ508, specifically at positions 184, 212, 276, 311, 335 and 347, were found (Figure 4(A,B)). As shown in Figure 4(B), the positions 184 and 212 are located in the N-terminal endonuclease and PA-linker of the PA protein, respectively, while the positions 276, 311, 335, and 347 are within C-terminal. To identify the residue(s) in PA that affect the polymerase activity, we generated two mutant plasmids based on pDP2002-JX198PA. pDP2002-JX198PA/ΔN containing mutants S184R and R212C, and pDP2002-JX198PA/ΔC containing mutants C276R, M311V, L335V, and G347D. The polymerase activity of the vRNP complex bearing pDP2002-JX198PA/ΔN was significantly higher than that of XZ508, whereas that of vRNP bearing pDP2002-JX198PA/ΔC was close to that of XZ508 (Figure 4(C)). To pinpoint which residue of the C-terminal of PA protein affects polymerase activity, four plasmids containing each amino acid mutation in the PA C-terminal of JX198 were generated, named as pDP2002-JX198PA/C276R, pDP2002-JX198PA/M311V, pDP2002-JX198PA/L335V, pDP2002-JX198PA/G347D, and polymerase activity of the vRNA bearing each of these PA mutants were tested. As described in Figure 4(D), the mutations in position 276, 311, and 335 had no effect on the polymerase activity, whereas the G347D mutation reduced the polymerase activity to levels close to that of XZ508. To further validate the critical role of the residue at position 347 of PA in regulating polymerase activity, we performed a reverse verification experiment by introducing the D347G mutation into the PA of XZ508. Functional assays demonstrated that the XZ508 PA D347G mutation significantly enhanced viral polymerase activity in JX198 backbone (Supplementary Figure S3). These data confirmed that the residue at position 347 of PA protein significantly affects the polymerase activity of JX198.

### D347G in PA affects viral vRNA synthesis

**Figure 5.**
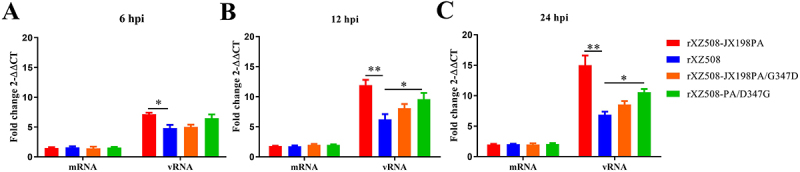
The relative NP gene viral vRNA and mRNA levels in MDCK cells infected with recombinant H9N2 viruses. The MDCK cells were infected with each recombinant virus at an MOI 1. NP-specific vRNA and mRNA levels were quantified by qRT-PCR at 6, 12, and 24 dpi. The *p* values were analyzed by using the student *t*-test. *p < 0.05; **p < 0.01; ***p < 0.001; ****p < 0.0001.

To determine the impact of residue 347 of PA on viral transcription and genome replication, a mutant containing single-point mutation in PA residue 347 of JX198 with XZ508 backbone, named as rXZ508-JX198PA/G347D, was rescued. The MDCK cells infected with rXZ508-JX198PA, rXZ508 and rXZ508-JX198PA/G347D, respectively, were collected at 6, 12, and 24 hpi, and the vRNA and mRNA levels of NP gene were quantified by qRT-PCR. No significant differences were observed in mRNA levels of NP in MDCK cells infected with these three viruses (Figure 5). However, the vRNA level of rXZ508-JX198PA was significantly higher than that of rXZ508 at 6, 12, and 24 hpi (*p* < 0.1, *p* < 0.01 and *p* < 0.01, respectively), whereas the mutation G347D significantly reduced the vRNA level of rXZ508-JX198 in MDCK cells (Figure 5). To further clarify the impact residue 347 of PA on viral transcription and genomic replication, we generated the XZ508 mutant containing the mutation D347G in PA (rXZ508 PA/D347G) and tested the vRNA and mRNA levels in infected MDCK cells. As shown in Figure 5, the D347G did not changed mRNA level of XZ508, but significantly increased the vRNA level of XZ508. To further verify the intracellular viral protein expression levels, Western blot assay were performed with infected cell samples collected at the same time points. The results showed the NP protein express levels were comparable among the four virus groups at each detected time point (6, 12 and 24 hpi), with noticeable differences in viral NP protein abundance between virus strains, which was highly consistent with the NP mRNA expression trends obtained from qRT-PCR analysis (Supplementary Figure S4). These data demonstrated that the residue 347 of PA protein mainly affects vRNA production, but not mRNA transcription in the virus life cycle.

### D347G in PA plays a key role in pathogenicity in mice

**Figure 6.**
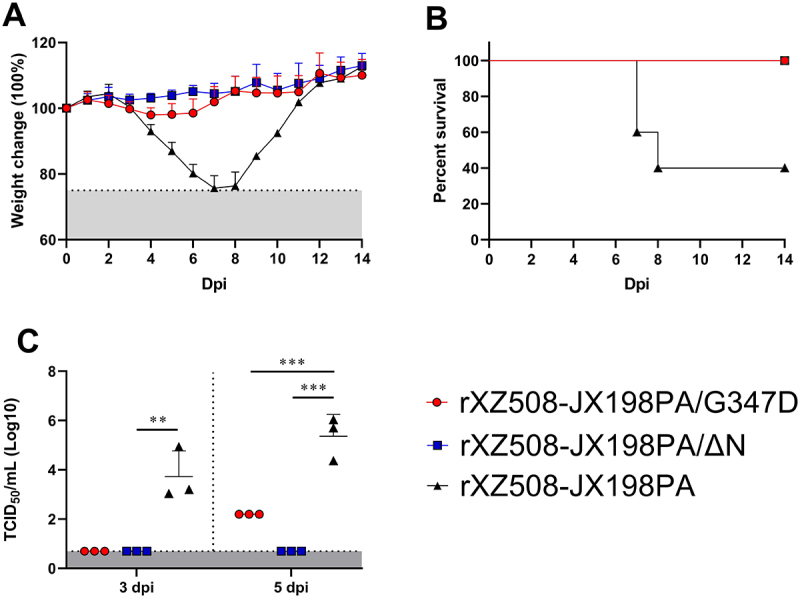
Replication and pathogenicity of rXZ508-JX198PA, and its PA mutants in BALB/c mice. 5-week-old BALB/c mice (11 mice/group) were infected with 10^5^ TCID_50_ of rXZ508-JX198PA, rXZ508-JX198 PA/G347D, and rXZ508-JX198 PA/ΔC. (A) Percentages of change in body weight and (B) survival following infection with each virus. (C) Virus titers in lung homogenates collected at 3 and 5 dpi were measured by TCID_50_ in MDCK cells. The *p* values were analyzed by using the student *t*-test. *p < 0.05; **p < 0.01; ***p < 0.001; ****p < 0.0001.

To further investigate whether mutation(s) in the C-terminate of PA contribute to the pathogenicity of JX198 in mice, we generated a mutant by introducing the mutations in C-terminate of PA of JX198 with XZ508 backbone, designated as rXZ508-JX198PA/ΔC containing mutations C276R, M311V, L335V, and G347D. The replication and pathogenicity of rXZ508-JX198PA/ΔC and rXZ508-JX198PA/G347D were evaluated in mice. Mouse study showed that the mice infected with rXZ508-JX198PA/ΔC showed no weight loss and no any clinical signs, and minor weight loss was observed in the rXZ508-PAJX198/G347D infected mice from 2 dpi and recovered from 7 dpi (Figure 6(A)). All the mice infected with rXZ508-JX198PA/ΔC and rXZ508-PAJX198/G347D survived by 14 dpi (Figure 6(B)). In contrast, the bodyweight of mice infected with XZ508-JX198PA declined rapidly from 3 dpi, and 3 out of the 5 mice had succumbed to the infection (Figure 6(A,B)). At 3 dpi and 5 dpi, 3 mice of each group were euthanized and viral titers in the lungs were determined. As shown in Figure 6(C), no virus was detected in the lungs from mice infected with rXZ508-JX198PA/ΔC, and low titers of virus in lungs from mice infected with rXZ508-PAJX198/G347D could be detected on 5 dpi (Figure 6(C)). In contrast, rXZ508-JX198PA could be efficiently detected with higher virus titers as described in Figure 6(C). These results demonstrated that the mutations in C-terminal of PA, especially D347G mutation, contribute to the pathogenicity of JX198 in mice.

### D347G in PA of JX198 affects viral plaque phenotype

**Figure 7.**
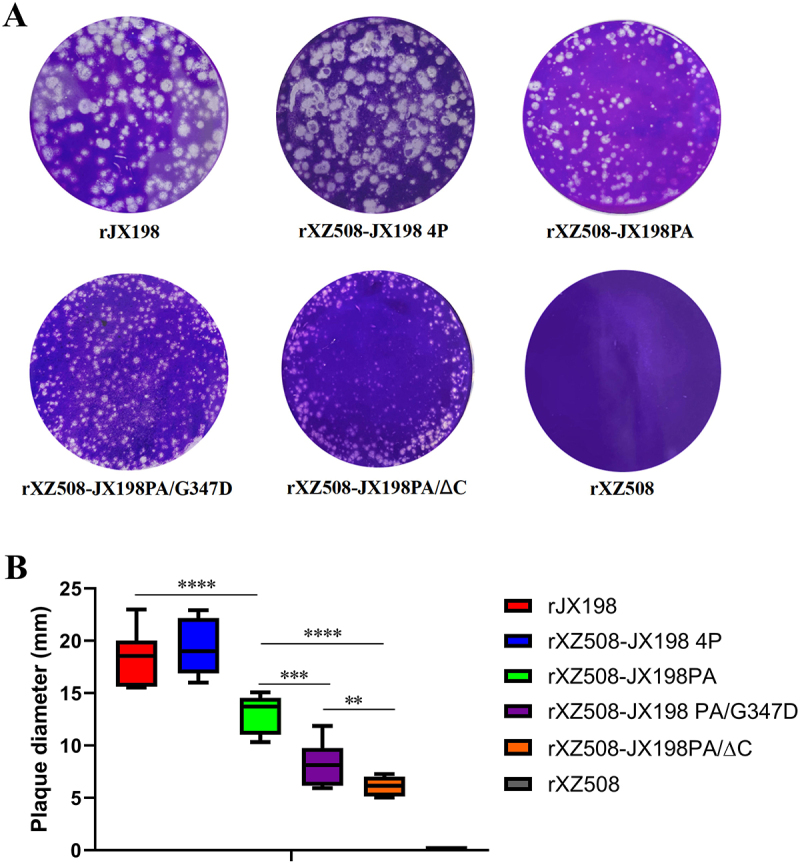
The plaque phenotype of the reassortants and mutants of XZ508. The plaque phenotypes of rJX198, rXZ508-JX198(4P), rXZ508-JX198 PA/G347D, rXZ508-JX198 PA/ΔC, and XZ508 were assessed by plaque assay on MDCK cells under a 1% agarose overlay. (A) Representative images of the plaque size of the reassortants and mutants of XZ508. (B) All plaque assays were performed under the same conditions, and the average plaque diameter of randomly selected ten plaques for each virus was measured and calculated using ImageJ software analysis. The *p* values were analyzed by using the student *t*-test. *p < 0.05; **p < 0.01; ***p < 0.001; ****p < 0.0001.

To investigate whether PA and specific residue mutations in PA of JX198 can affect the viral plaque phenotype, rJX198, rXZ508-JX198 4P, rXZ508-JX198PA, rXZ508-JX198PA/ΔC, rXZ508-JX198PA/G347D, and rXZ508 were selected for testing. As shown in Figure 7, rJX198 and rXZ508-JX198 4P produced significantly larger plaques, whereas rXZ508 did not exhibit discernible plaque phenotype. Although XZ508-JX198PA presented smaller plaques than that of rJX198 and rXZ508-JX198 4P, it also had a clear plaque phenotype. The plaque sizes formed by rXZ508-JX198PA/ΔC and rXZ508-JX198PA/G347D were further reduced (Figure 7). All these demonstrated that the PA of rJX198, especially D347G mutation, affects viral plaque phenotype.

## Discussion

The H9N2 virus is widespread across most parts of Asia, the Middle East, and North Africa [9]. It not only causes severe economic losses to the poultry industry, but also can jump from avian species to mammalian species, including humans [31,32], which raises concerns about its potential to cause a pandemic. In this study, we focused on two H9N2 viruses isolated from chickens that these two viruses have different phenotypes in mice: XZ508 is nonpathogenic, whereas JX198 is highly lethal in mice. Through the generation and testing of a series of reassortant and mutant viruses, we demonstrated that PA is the major virulence factor of JX198, and the amino acid at position 347 of PA is the key determiner that can increase pathogenicity in mice and polymerase activity.

The pathogenicity and transmissibility of avian IAVs in mammals are influenced by different factors that are not fully understood. HA and vRNP are considered to be the two most important factors influencing the pathogenicity of IAVs. Some residue mutations in HA have been shown to alter its preference for human-type receptors, and thereby increasing the transmissibility of avian IAVs, especially Q226L and G228A/S (H3 numbering), which can increase the number of subtypes of avian IAVs binding to human-type receptors, replication in mammalian cells, and virulence and transmission in mammals [33–35]. Amino acids 627K and 701N in PB2 have also been reported to increase the pathogenicity of several subtypes of avian IAVs in mice and ferrets [5,36,37]. In this study, we found that the major factor driving the high lethality of the H9N2 virus in mice was the D347G mutation acquired in PA, rather than the 627K mutation in PB2. While this observation implies that AIVs may employ diverse molecular mechanisms to enhance pathogenicity in mammals, however, this hypothesis remains to be fully validated by future studies that systematically test the independent and combinatorial effects of non-PA gene mutations.

Notably, PB2 and PB1 from JX198 could enhance viral pathogenicity in mice when reassorted into the XZ508 backbone, but this *in vivo* virulence enhancement was not accompanied by a corresponding increase in polymerase. This inconsistent result might be due to the fact that the minigenome assay only measures the polymerase activity of the vRNP *in vitro*, while viral pathogenicity in mice is a complex phenotype involving tissue‑specific viral replication, host innate immune modulation, and viral spread efficiency. We further compared the amino acid sequences of PB2, PB1, and NP between JX198 and XZ508: 5 amino acid variations in PB2 (T76A, K105T, V292I, V338I, V613I), 16 amino acid variations in PB1 (A14V, N52K, A56T, I69V, S173T, I200V, R214K, R235K, M317V, M372I, K383Q, I525V, A573T, P598L, D619E, S642N), and 4 amino acid variations in NP (A2V, M136L, G391R, and R452K). Among these mutations, PB2 V292I has been shown to be associated with the enhancement to the polymerase activity of AIV in mammals and pathogenicity in mice [38]. Whether PB2 V292I and other previously uncharacterized amino acid mutations contribute to the increased pathogenicity of JX198 in mice requires further investigation.

In addition of PB2, PB1, and PA, our data also revealed that the NP gene of JX198 moderately contributed to viral pathogenicity in mice (Figure 2). Moreover, the polymerase activity assays further revealed that NP gene from JX198 significantly enhanced the vRNP polymerase activity of XZ508 (Figure 3). NP of influenza virus is an essential component vRNP complex that participates in viral RNA encapsulation and replication [39]. The four amino acid variation (A2V, M136L, G391R, and R452K) identified between JX198 and XZ508 NP may affect vRNP stability, RNA-binding affinity, or protein-protein interaction within the replication complex, thereby affecting viral fitness in mice. Further studies are required to clarify whether these NP substitutions individually or cooperatively contributed to enhance polymerase activity, viral replication, or mammalian adaptation.

The distribution of residues at position 347 of PA of H9N2 viruses circulating in chickens in China was analyzed, the results showed that G347 is rare (0.53%), and more than 99%, even 100%, of H9, H5, and H7 viruses circulating in chickens in China contain D at this position (Table 2). However, it should be noted that only a few PA sequences of avian IAVs circulating in chickens in China have been included in the GISAID and NCBI databases, particularly after 2020. Therefore, the currently available datasets may not fully reflect the recent evolutionary dynamics or the true prevalence of this mutation in circulating H9N2 viruses.

**Table 2.** Amino acid diversity of PA position 347 of avian IAVs circulating in chickens in China.

Viruses	D (%)	G (%)	Other (%)
H9N2	99.19	0.54	E (0.18), N (0.09)
H5Ny	100	0	0
H7Ny	100	0	0

The PA protein of avian IAV is the third protein of vRdRp and contains 716 amino acids. It is composed of the N-terminal endonuclease region and the larger C-terminal region connected by a PA linker [40,41], which also plays a crucial role in the adaptation and pathogenicity of avian IAVs in mammals. The residue at PA position 347 is located at the edge of the PA C-terminal endonuclease region (Figure 4(A)), which has been shown to affect viral polymerase activity and the pathogenicity of H5N1 and H7N9 viruses in mice [42,43]. In previous studies on generating H9N2 mouse adaptation strains, it was showed that G347D of PA can increase polymerase activity and virulence [26,27]. However, these studies only characterized this mutation in mouse adapted H9N2 strains generated by serial passaging in mice. Our study identified that the PA D347G is a naturally occurring mutation in a field G57 genotype H9N2 isolate, and this mutation is sufficient to confer high pathogenicity in mice without prior adaptation. This key distinction highlights the direct and immediate zoonotic risk of naturally circulating H9N2 strains carrying the PA D347G mutation are already present in poultry populations, which is an important consideration for on-field surveillance and risk assessment that was not addressed in previous mouse-adaptation studies.

Han et al. reported that the vRdRp of human influenza A/NT/60/1968 (H3N2) and avian influenza A/duck/Fujian/01/2002 (H5N1) forms dimers of heterotrimers with dimerization mediated by the C-terminal domain of the PA subunit, the thumb subdomain of PB1 and the N1 subdomain of PB2, which plays an important role in vRNA synthesis during replication of the viral genome [40]. Structural analyses revealed that PA residue 347 is positioned at the polymerase dimer interface and participates in hydrogen-bond interaction with PB2 residues 76T and PB2 70 R [40]. Therefore, substitution of 347D with glycine may alter the stability or dynamics of polymerase dimer formation. 347D contains a carboxylic acid side chain and a negatively charged functional group, which helps it to form the hydrogen bridge with other amino acids. However, 347G lacks both the side chain length and charge required for these interactions. Interestingly, our results showed that PA D347G significantly enhanced vRNA accumulation without affecting viral mRNA levels (Figure 5). This observation is consistent with the notion that influenza virus transcription and genome replication are mechanistically distinct processes. Viral mRNA synthesis primarily depends on the cap-snatching endonuclease activity located within the N-terminal domain of PA, whereas vRNA synthesis requires coordinates assembly and dimerization of the polymerase complex [4,44]. Therefore, the D347G substitution is unlikely to directly influence transcription initiation but may alter the efficiency or dynamics of polymerase dimer formation during genome replication, thereby preferentially promoting vRNA synthesis. The precise molecular mechanism of PA residue 347 represents a critical determinant regulating vRNA replication remains to be further investigated.

It is important to acknowledge the experimental limitations of the present study. First, all in mouse challenge studies were conducted with a single high inoculation dose of 10^5^ TCID_50_/mouse, which was achieve the primary objective, to clearly distinguish the pathogenic phenotypes between JX198 and XZ508 and to directly the molecular determinants of the observed lethality in mice. Nevertheless, the inclusion of low-dose inoculation data and MLD_50_ determination would further quantify the contribution of the PA D347G mutation to viral virulence, provide nuanced insights into the dose-response relationship between viral inoculum and pathogenicity in mice, and potentially reveal subtle differences in viral replication or host immune modulation that may not be apparent at a high challenge dose. Additionally, all functional experiments investigating the effect of PA D347G were performed using the XZ508 backbone, a G57 genotype H9N2 strain that is the dominant lineage in China; while our findings are highly relevant for this prevalent genotype, the effect of PA D347G on viral pathogenicity may be genotype- or strain-dependent, and whether this mutation confers similar enhancements in other H9N2 genotypes or non-H9N2 avian IAVs remains to be investigated. Finally, reciprocal reassortant experiments using JX198 as the backbone were not performed. However, as shown in Figure 2, all four vRNP components (PB2, PB1, PA, and NP) derived from JX198 individually boost viral pathogenicity in mice. Even if we reverted the G347 residue to D within the PA of JX198, the remaining virulence factors harbored by PB2, PB1, and NP may retain substantial pathogenic potential in mice. The contribution of PA G347D may be partially obscured by additional determinants in PB2, PB1, and NP, resulting in ambiguous experimental readouts that cannot deliver clearer or more definitive evidence compared with the XZ508 backbone system.

Understanding the molecular basis of the increased fitness of avian IAVs in both avian and mammals is essential for assessing the risks these viruses pose to food industry and public health. In this study, we have revealed that the PA D347G mutation is the key mutation for increased pathogenicity of the H9N2 virus in mice. Our work may provide guidance for future surveillance of avian IAVs and may help us to better understand the molecular basis of viral fitness and virulence in mammals.

## Supplementary Material

S Figure 4.jpg

S Figure 3.jpg

S Figure 2.jpg

S Figure 1.jpg

## Data Availability

The data that support the findings of this study are openly available in Mendeley Data at D OI: 10.6084/m9.figshare.28103315 [45].
