# Supplementary figures and images for: D347G in PA is critical for the pathogenicity of H9N2 avian influenza A virus in mice
Source: Virulence. 2026 Aug 2;17(1):2711518. doi: 10.1080/21505594.2026.2711518 (PMC13432829; doi:10.1080/21505594.2026.2711518)

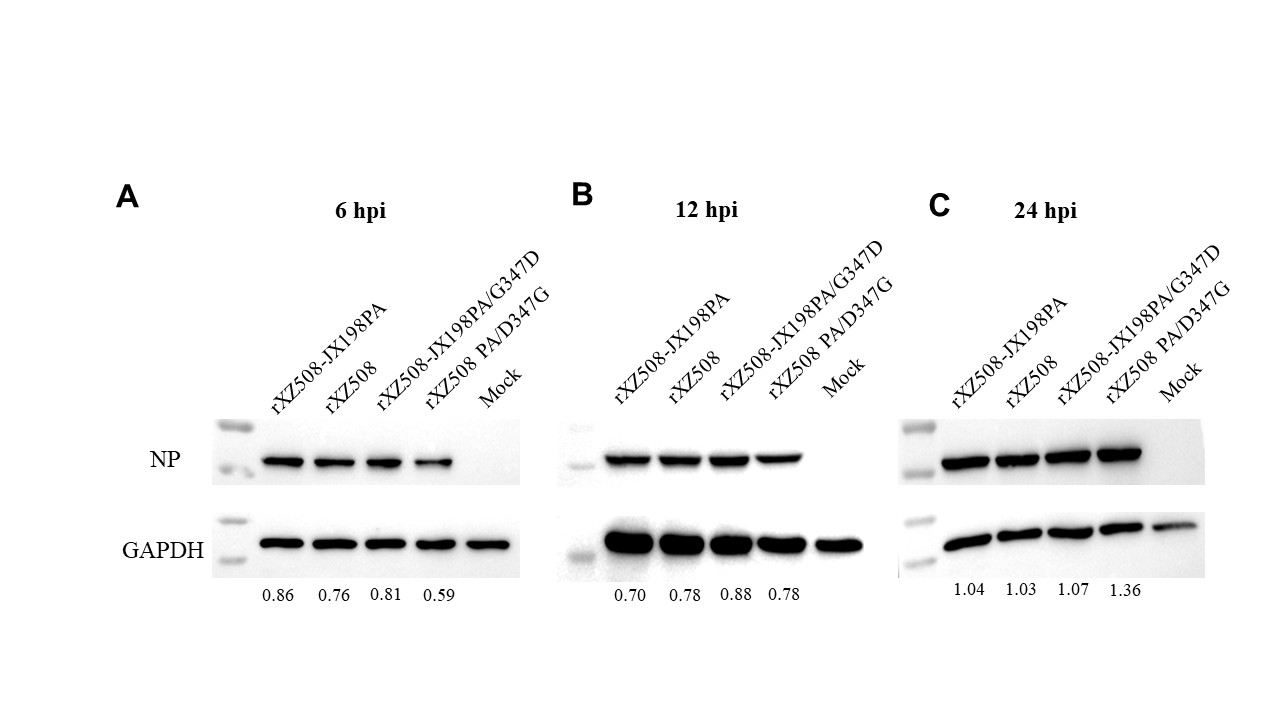

Supplement: S Figure 4.jpg [file KVIR_A_2711518_SM9812.jpg]

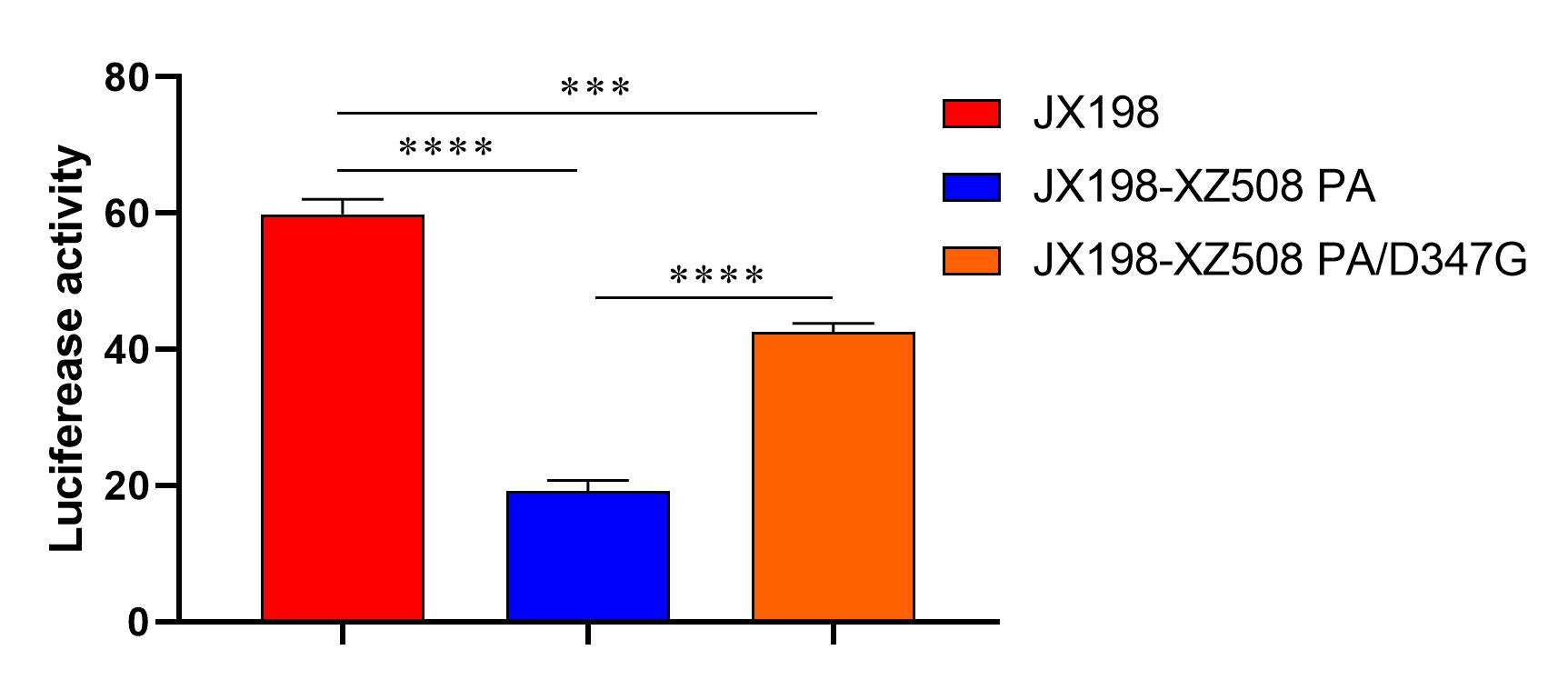

Supplement: S Figure 3.jpg [file KVIR_A_2711518_SM9811.jpg]

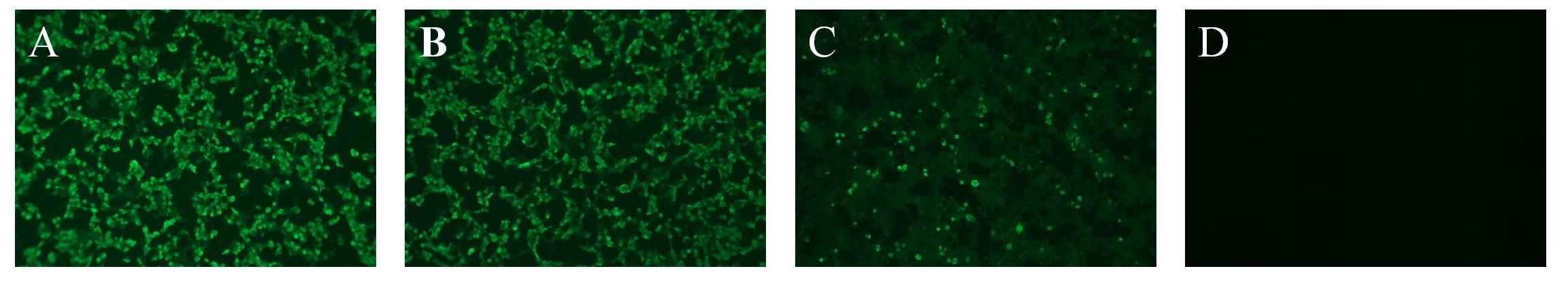

Supplement: S Figure 2.jpg [file KVIR_A_2711518_SM9810.jpg]

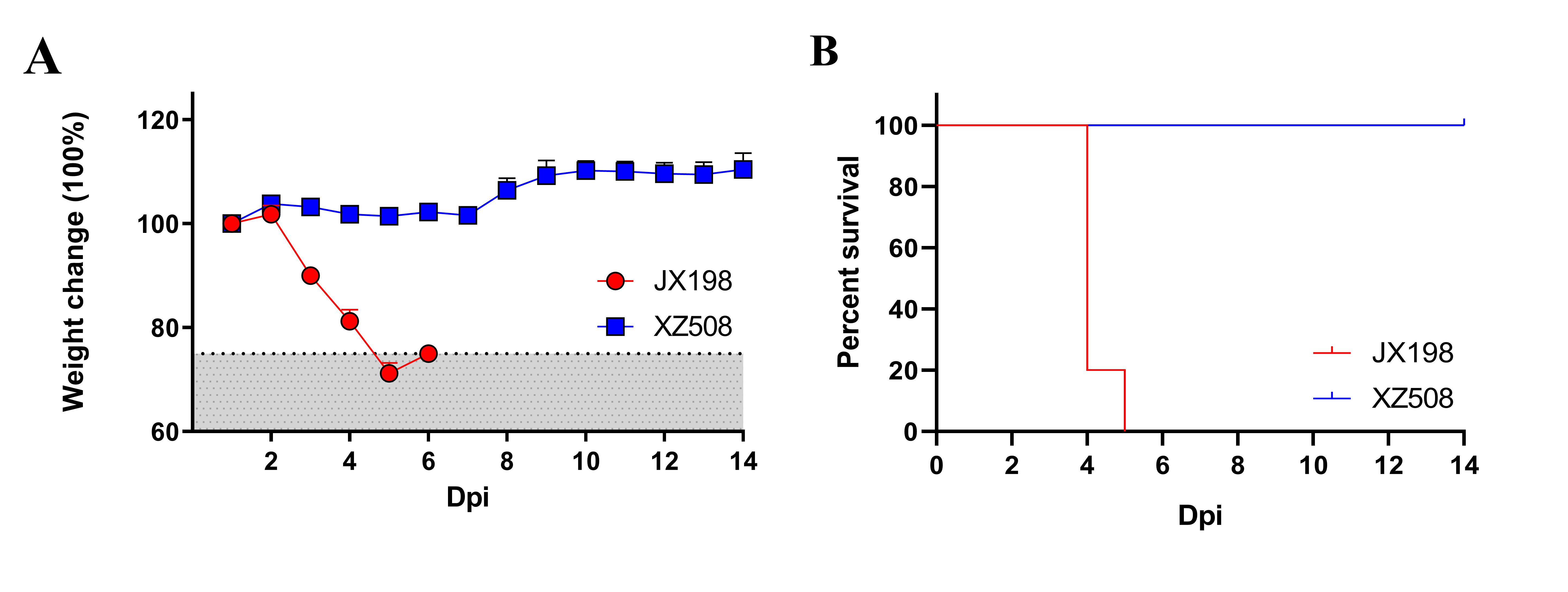

Supplement: S Figure 1.jpg [file KVIR_A_2711518_SM9809.jpg]
